# Palliative care assessment of dry mouth: what matters most to patients with advanced disease?

**DOI:** 10.1007/s00520-019-04908-9

**Published:** 2019-06-14

**Authors:** Michelle Fleming, Cheryl L. Craigs, Michael I. Bennett

**Affiliations:** Academic Unit of Palliative Care, Leeds Institute of Health Sciences, University of Leeds, Leeds, LS2 9JT UK

**Keywords:** Dry mouth, Xerostomia, Palliative care, Advanced disease, Assessment, Cross-sectional study

## Abstract

**Purpose:**

Dry mouth is a highly prevalent and significant symptom in patients with advanced progressive diseases. It is a poorly understood area of research, and currently, there is no standardised outcome measure or assessment tool for dry mouth.

**Methods:**

To assess responses to self-reported dry mouth questions, the impact of dry mouth, methods used to reduce symptoms and relevance of the questionnaire. A cross-sectional multisite study of 135 patients with advanced progressive disease experiencing dry mouth. Participants were located in the inpatient, day care, outpatient or community setting.

**Results:**

The majority (84.4%) of patients rated their dry mouth as moderate or severe using the verbal rating scale (VRS). Seventy-five percent (74.7%) had a numeric rating scale (NRS) score of 6 or more for dry mouth severity. Patients reported that dry mouth interfered most with talking and was the most important function to assess (median score 6 out of 10) followed by eating (median 5) and taste (median 5). Taking sips of drink was the most common and most effective self-management strategy. Over half of patients (54.6%) also reported impact on swallow and sleep and associated dryness of lips, throat and nasal passages.

**Conclusions:**

This study highlights the severity of dry mouth in advanced disease. Important factors when assessing patients with dry mouth should include the functional impact on day-to-day activities including talking, dysphagia and sleep. Simple considerations for patients include provision of drinks and reviewing medications. This study could be used to develop a standardised assessment tool for dry mouth to use in clinical practice.

## Introduction

Dry mouth is a highly prevalent and significant symptom for patients with advanced progressive diseases. The estimated prevalence of dry mouth in the general population is between 21 and 27% [1]. In a UK study of 197 terminally ill cancer patients, it was the most prevalent symptom, occurring in 77% [2]. Dry mouth was described as the ‘orphan topic in supportive care’ in 1997 [3], as, up until this time, the research in dry mouth was largely in healthy patients, those with Sjogren’s syndrome, or in those who had received radiotherapy to the head and neck.

One of the first studies on the epidemiology, aetiology and clinical features of dry mouth in advanced cancer patients was published in 2001 [4]. In this study, 78% of 120 patients reported dry mouth; it was the fourth most common symptom reported on a Memorial symptom assessment scale (MSAS) and the 3rd most distressing symptom. The majority (75%) of the patients reported by Davies [4] experienced dry mouth either ‘frequently’ or ‘almost constantly’, and 86% rated the severity as either ‘moderate’, ‘severe’ or ‘very severe’.

Saliva has a number of key functions, the most important of which is to lubricate food prior to swallowing. Saliva consists of more than 99% water, and along with secretions containing mucin glycoproteins, it helps to coat, lubricate and protect the hard and soft surfaces of the oral cavity [5–7]. It contains amylase for digestion of starch and lingual lipase for fat digestion [5]. Saliva has an antibacterial function, containing lysozymes that break down bacterial cell walls [5, 7]. The salivary flow is increased by mastication, and this allows for bolus formation and swallowing. Saliva triggers thirst perception and enhances taste perception [5]. Dry mouth can result in functional difficulties such as difficulty chewing, difficulty swallowing (dysphagia) and taste disturbance (dysgeusia). Persistent dry mouth also predisposes to oral infections, dental caries and oral candidiasis [5, 8]. Furthermore, patients report dry mouth as a distressing symptom with a significant impact on their quality of life [9].

Drug treatment is the most common cause of dry mouth in the general population [10]. Patients with advanced diseases are often prescribed a large number of medications, many of which have recognised anticholinergic effects, such as opioids, corticosteroids and benzodiazepines [11], and cannot be discontinued due to refractory symptomatology. ‘Anticholinergic load’ has been recently studied in a retrospective review of patients with advanced diseases at the end of life. In 199 patients, 60% received anticholinergic medication in the last 72 h of life [12].

There are few treatments licenced for use to help this condition. A systematic literature review on the management of dry mouth in advanced cancer patients identified only three randomised controlled trials and three prospective studies [13]. Overall, randomised controlled trials support the use of pilocarpine, artificial saliva and chewing gum for the management of xerostomia [14–16]. However, the evidence is of low quality.

Dry mouth in patients with advanced progressive diseases is a poorly understood area of research. Studies reporting on the assessment of dry mouth and outcomes of interventions have used varying scales and tools [14–18]. Some researchers used additional symptoms other than dry mouth such as dysphagia, dysgeusia and dysarthria. Secondary outcomes have included the use of additional questionnaires relating to overall opinion and preference to continue treatment [14, 15]. A more recent study has used validated tools from non-palliative care populations [18]. The Memorial symptom assessment scale (MSAS) and the Edmonton symptom assessment scale (ESAS) are validated symptom assessment tools commonly used in the palliative care population, but they are not specific for dry mouth. There is no consensus on the most valid approach that also incorporates the functional impact on the patient. To our knowledge, there is no validated dry mouth assessment tool for the palliative care patient.

## Aim

The aims of this study were to assess, in a population of palliative care patients with dry mouth, responses to different measures of self-reported dry mouth questions; the impact of dry mouth on day-to-day activities such as eating, talking and taste; and methods used to reduce symptoms. A secondary aim was to assess what parts of the questionnaire patients found most relevant to their dry mouth.

## Methods

### Study design

This is an exploratory cross-sectional study designed to assess the important factors in the clinical assessment of dry mouth for the palliative care patients using questionnaires administered face-to-face. The study received NHS Research Ethics Committee approval on 12 October 2015 (IRAS Project ID 182067).

### Setting

Recruitment took place over four sites. Three hospices (palliative care inpatient units) and one large teaching hospital all based within the north of England were included. Recruitment took place between 1 December 2015 and 30 November 2016.

### Participants

Eligible patients were at least 18 years of age with advanced disease who had either been referred to palliative care services or had been identified as being within the last year of life were identified as having dry mouth, either by clinical staff or self-reported during clinical assessment, and who had the ability to consent to the study. All patients were included irrespective of the cause of their dry mouth. Participants were located in the inpatient, day care, outpatient or community setting.

Eligible patients were provided with information on the study, and those who consented to take part were included in the study. Written consent was obtained from all participants.

### Questionnaire

The questionnaire incorporated assessment tools used to assess dry mouth in palliative care, identified through a literature search, and clinical questions used in routine practice. Patients included in the study were taken through the questionnaire by the researcher face-to-face, and responses were recorded by the researcher. Interviews took no longer than 15 min to complete.

The questionnaire contained questions covering the following:

### Dry mouth severity

Patients were asked to respond to the question ‘Does your mouth usually feel dry?’ using a dichotomous response, responding ‘Yes’ or ‘No’. Patients were then asked ‘Tell me about your dry mouth’ using the following 2 scales:

- A numeric response using a numeric rating scale (NRS) between 0 and 10, with 0 representing ‘No dryness’ and 10 representing ‘Worst imaginable dryness’.
- A descriptive response using a verbal rating scale (VRS) including the categories ‘None’, ‘Mild’, ‘Moderate’ or ‘Severe’.

### Interference

Patients were asked about the impact of dry mouth on talking, eating and taste using a numeric rating scale (NRS) of 0–10, with 0 representing ‘No interference’ and 10 representing ‘Significant interference’. The three questions were:

- Does your dry mouth interfere with talking?
- Does your dry mouth interfere with eating?
- Does your dry mouth interfere with taste?

### Self-management

Patients were asked if they needed to use things to keep their mouth moist and were prompted to report, using free text, what these things were.

### Other concerns

Patients were asked to respond ‘Yes’ or ‘No’ to the question:

‘Is there something not already mentioned that is of particular concern as a result of dry mouth? When patients responded ‘Yes’, concerns were recorded as free text.

### Question assessment

Patients were asked three additional questions to identify the items in the questionnaire which they felt ‘best sums up their dry mouth’, they ‘found easiest to complete’ and they ‘found hardest to complete’.

### Patient characteristics

Patient’s demographic characteristics of age, gender, diagnosis, the commonly used clinical assessment domains of illness phase (Stable, Unstable, Deteriorating or Terminal) and performance status (recorded using either the Karnofsky performance status or the Barthel score) and drug history were obtained from the clinical notes. The Barthel Index is an objective tool [19] using a sum score (0–100) across ten domains which assesses an individual’s ability to perform activities of daily living. It has been used in patients with advanced illness and in terminally ill hospice patients, a rate of change in Barthel score has been correlated with survival [20]. The Karnofsky performance scale (KPS) and more recently the Australian Karnofsky performance scale (AKPS) assesses functional performance on an 11-point scale from 0 to 100% and is important in resource utilisation in palliative care [21]. The total anticholinergic load of the patient’s medication was recorded using an anticholinergic drug scale (ADS) [22].

### Statistical analysis

Categorical patient characteristics and responses to the dry mouth questionnaire were summarised using frequencies (*n*) and percentages. Responses to numeric rating scales in the dry mouth questionnaire were summarised using the median and inter-quartile range (IQR), for skewed data, and the mean, and standard deviation (SD) for non-skewed data.

The association between dry mouth severity NRS and performance status was assessed using the Spearman’s rank correlation coefficient. Differences in dry mouth severity NRS between categorical variables were compared using the Mann-Whitney *U* test, for two group comparisons, or the Kruskal-Wallis *H* test, for more than two groups.

Data was analysed using SPSS statistical software (version 22-24). A two-tailed *p* value of less than 0.05 was used to define statistical significance.

## Results

Table 1 provides a summary of patient characteristics.

**Table 1:** Patient characteristics

Patient characteristics	*n*	Column %
Total	135	100.0
Gender		
Male	53	39.3
Female	82	60.7
Age groups		
Mean (SD)	71.2	(11.2)
Primary diagnosis		
Breast	12	8.9
Lung	25	18.5
Colorectal	10	7.4
Other malignancy	57	42.2
Non-malignancy	31	23.0
Stage of illness		
Stable	61	45.2
Unstable	44	32.6
Deteriorating	27	20.0
Terminal	2	1.5
Missing	1	0.7
Karnofsky performance status (10–100)		
Count	56	
Mean (SD)	54.8	(19.2)
Barthel Index (0–100)		
Count	78	
Mean (SD)	71.3	(21.7)
Total anticholinergic load		
0	1	
1–12	134	
Mean (SD)	3.9	(2.4)
Does your mouth usually feel dry?		
Yes	112	83.0
No	21	15.6
Missing	2	1.5
Severity of current dry mouth: verbal rating scale (VRS)		
None	0	0.0
Mild	21	15.6
Moderate	67	49.6
Severe	47	34.8
Severity of current dry mouth: numerical rating scale (NRS, 0–10)		
0	0	0.0
1	0	0.0
2	3	2.2
3	1	0.7
4	7	5.2
5	23	17.0
6	17	12.6
7	23	17.0
8	30	22.2
9	13	9.6
10	18	13.3
Median		7
IQR		3 (5 to 8)

### Patient demographics

One hundred thirty-five patients completed the questionnaire.

Over 60% (60.7%) were female. The age of patients ranged from 32 to 92 years, with a mean age of 71 years (SD 11.2). Seventy-seven percent (77%) had a diagnosis of malignancy; lung cancer was the most common malignancy (18.5%) followed by breast cancer (8.9%) and colorectal cancer (7.4%). COPD was the most common non-malignant disease and accounted for 12% of participant’s diagnoses.

Over half of patients (57.8%) had a Barthel Index score recorded, with the remainder assessed using the Karnofsky performance status; one patient had been assessed using both measures. Only one patient was not taking medications that had anticholinergic activity. The mean anticholinergic load score on the ADS was 3.9 (SD 2.4). Typical medications included opioids, corticosteroids, anti-emetics and benzodiazepines. Though all patients were identified as having a dry mouth, just over 15% (15.6%) of patients reported that their dry mouth did not usually feel dry using the dichotomous scale. Almost half (48%) of those reported that the dry mouth was a new or recent occurrence during their hospital admission.

### Dry mouth severity

Most patients reported that they usually had a dry mouth (*n* = 112, 83.0%).

Patients reported on the current severity of their dry mouth, using the VRS and NRS scales. The lowest VRS rating for usual dry mouth was ‘Mild’, with 84.4% of patients rating their current dry mouth as either moderate or severe. The lowest NRS rating was 2 out of 10, with a median current dry mouth severity of 7 (IQR = 3). Seventy-five percent (74.7%) had a NRS score of 6 or more.

**Fig. 1:**
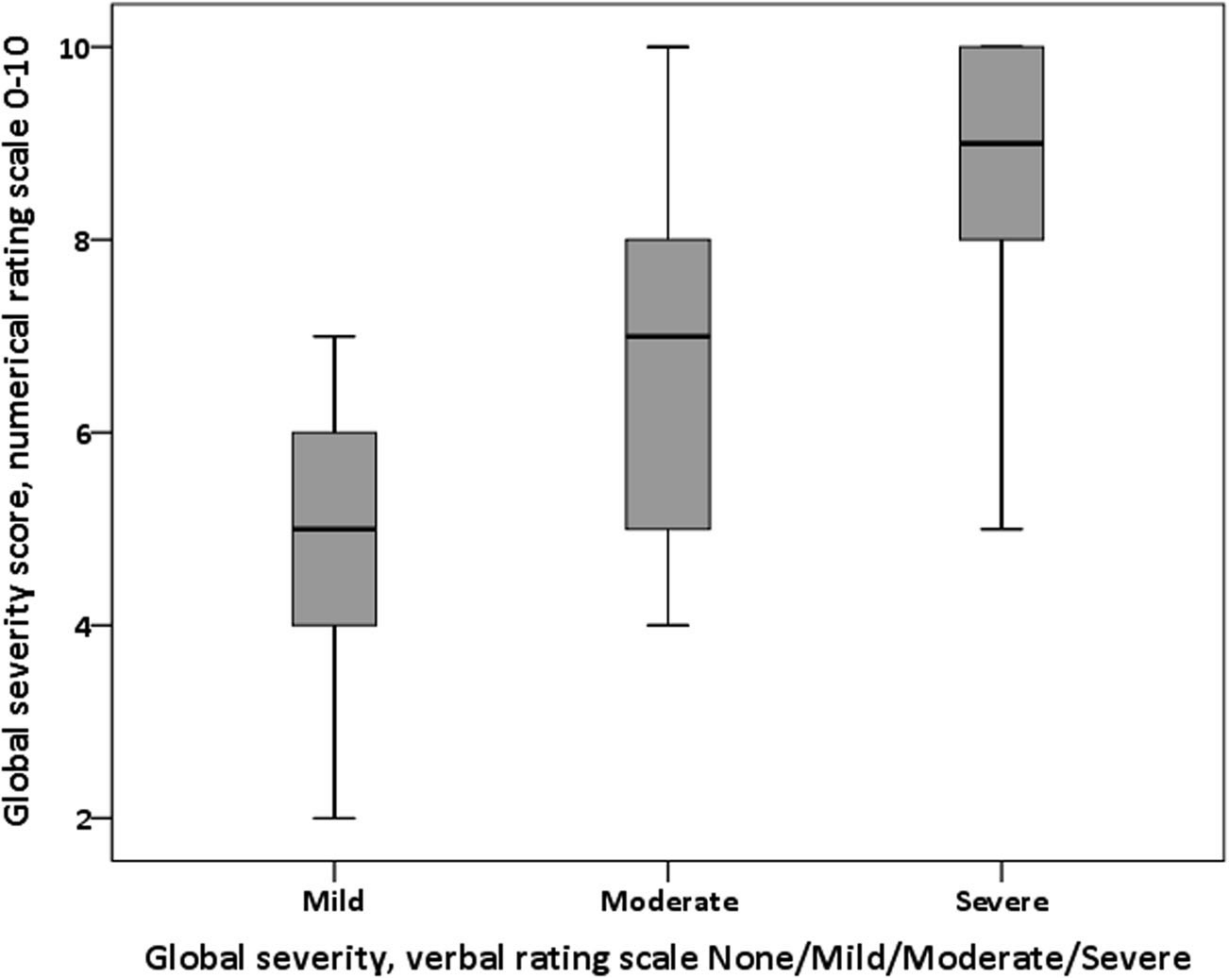
Distribution of dry mouth NRS responses by VRS

There was a significant positive relationship between the distribution of responses to the NRS dry mouth severity score and the responses to the VRS dry mouth severity score (Kruskal-Wallis = 63.10, df = 2, *p* < 0.001), suggesting consistency between dry mouth severity scoring using the two scales. Median scores increased with each VRS category increase (mild: median = 5, IQR = 2; moderate: median = 7, IQR = 3; severe: median = 9, IQR = 2), though there was overlap in NRS responses between VRS responses (Fig. 1).

### Interference with talking, eating or taste

Table 2 shows the distribution of responses to the questions relating to how much dry mouth interferes with talking, eating or taste. Most patients reported some (response 1 or more out of 10) interference with talking (75.6%), eating (61.2%) or taste (59.7%), with only 13.3% of patients (*n* = 18) reporting no interference for all of these three measures. Patients reported a median score of 6 out of 10 (IQR 7) for interference with talking, and 5 out of 10 for interference with eating (IQR 7) or taste (IQR 5).

**Table 2:** Patient responses to interference with talking, eating or taste, measured on a 0–10 scale, by severity of current dry mouth, measured using the verbal rating scale (VRS)

Interference with:	VRS response	(Response = 0) no interference		(Response > 0) some interference		Median	IQR
		*n*	Row %	*n*	Row %		
Talking							
Mild		10	47.6	11	52.4	1	4 (0 to 4)
Moderate		19	28.4	48	71.6	5	7 (0 to 7)
Severe		4	8.5	43	91.5	8	2 (7 to 9)
Total		33	24.4	102	75.6	6	7 (1 to 8)
Eating							
Mild		12	57.1	9	42.9	0	5 (0 to 5)
Moderate		29	43.3	38	56.7	4	7 (0 to 7)
Severe		11	23.9	35	76.1	7	7 (0.75 to 8)
Total		52	38.8	82	61.2	5	7 (0 to 7)
Taste							
Mild		10	47.6	11	52.4	2	5 (0 to 5)
Moderate		32	47.8	35	52.2	3	7 (0 to 7)
Severe		12	26.1	34	73.9	6.5	8 (0 to 8)
Total		54	40.3	80	59.7	5	7 (0 to 7)

The distribution of responses differed significantly for current dry mouth responses using VRS with the level of reported interference increasing with the severity of dry mouth reported (talking: K-W = 39.13, *p* < 0.001; eating: K-W = 15.94, *p* < 0.001; taste: K-W = 11.58, *p* = 0.003).

Current severity of dry mouth reported on the NRS had significant positive relationships with interference with talking, eating and taste (talking: *r*_s_ = 0.491, *p* < 0.001; eating: *r*_s_ = 0.338, *p* < 0.001; taste: *r*_s_ = 0.282, *p* = 0.001) (Table 3).

**Table 3:** Association between responses to the dry mouth numerical rating scale (NRS) and interference with talking, eating and taste

Numerical rating scale (NRS)	Talking	Eating	Taste
*β* coefficient	0.77	0.57	0.51
Standard error of *β* coefficient	0.13	0.15	0.16
Spearman’s rank correlation (*r*_s_)	0.49	0.34	0.28
*p* value	< 0.001	< 0.001	0.001

### Self-management

Almost all patients (*n* = 131, 97.0%) reported that they needed interventions to help keep their mouth moist. Table 4 shows the types of items patients reported using to keep their mouth moist. Of the patients who reported using items for moisture, 64.6% reported using more than one (Table 4). Multiple items included a combination of non-pharmacological aids such as drinks, sweets and mouthwashes and pharmacological aids such as salivary substitute sprays and gels. Almost all (93.4%) of the patients who reported using single options used drinks alone. Other methods reported were toothpaste and chewing gum.

**Table 4:** Items reported to help keep the mouth moist

Dry mouth response	Drinks		Sweets		Other		Multiple		Total
	*n*	Row %	*n*	Row %	*n*	Row %	*n*	Row %	
Total	43	33.1	1	0.8	2	1.5	84	64.6	130
Verbal rating scale (VRS)									
Mild	8	38.1	0	0.0	1	4.8	12	57.1	21
Moderate	23	36.5	0	0.0	1	1.6	39	61.9	63
Severe	12	26.1	1	2.2	0	0.0	33	71.7	46
Numerical rating scale (NRS)									
2	1	33.3	0	0.0	0	0.0	2	66.7	3
3	1	100.0	0	0.0	0	0.0	0	0.0	1
4	4	57.1	0	0.0	0	0.0	3	42.9	7
5	6	30.0	0	0.0	1	5.0	13	65.0	20
6	9	52.9	0	0.0	1	5.9	7	41.2	17
7	4	19.0	0	0.0	0	0.0	17	81.0	21
8	13	43.3	0	0.0	0	0.0	17	56.7	30
9	3	23.1	1	7.7	0	0.0	9	69.2	13
10	2	11.1	0	0.0	0	0.0	16	88.9	18

### Other concerns

Almost half of the patients (*n* = 65, 48.1%) responded ‘Yes’ to the question ‘Is there something not already mentioned that is of particular concern as a result of dry mouth’:

- 24.6% (*n* = 16) mentioned that their lips, throat or nasal passages were also dry.
- 15% (*n* = 10) of patients mentioned that it woke them up at night.
- 15% (*n* = 10) mentioned that their dry mouth affected their swallow and resulted in a change in diet, a reduction in intake of food and even drinks.
- 7.6% (*n* = 5) mentioned that they felt the cause may have been due to medications. A further 7 patients mentioned that medications were the reason for the new onset of dry mouth.

### Question assessment

Table 5 shows the question which patients thought best summed up their dry mouth. Interference with talking was the most popular question to sum up their dry mouth. The three questions on interference combined were identified by 44.4% (*n* = 60) of patients as including a question which best summed up their dry mouth, compared with 25.9% (*n* = 35) of patients who chose one of the severity scales.

**Table 5:** Responses to question: Which question/measure best sums up your dry mouth?

Response:	*n*	Column %
Dry mouth severity: dichotomous	3	2.2
Dry mouth severity: NRS	23	17.0
Dry mouth severity: VRS	9	6.7
Interference: talking	32	23.7
Interference: eating	11	8.1
Interference: taste	12	8.9
Interference: all 3 questions	5	3.7
Other concerns	2	1.5
Unsure/no preference	32	23.7
Missing	6	4.4
Total	135	100.0

## Discussion

Our study found that most patients with advanced disease that have previously been identified as having a dry mouth rated their dry mouth as moderate or severe using a VRS, or at least a 6 out of 10 on a NRS, for dry mouth severity. The responses patients provided for the VRS scale were consistent with the NRS scale.

Over 85% of patients reported that dry mouth interfered, at least to some extent, with talking, eating or taste. The extent to which dry mouth interfered was significantly related to the reported severity of dry mouth. Interference with talking due to dry mouth was the most common problem, with three quarters of patients reporting at least some interference with talking. Nearly one quarter of patients also identified this question as the measure which best summed up their dry mouth. Facilitating time for patients to have a drink during a consultation can ease this burden [23].

Correlation of dry mouth and impact on talking has been noted in previous studies [4, 14]. The study by Sweeney [14] of 35 hospice patients used visual analogue scale (VAS) scores at baseline and following administration of saliva spray or placebo. They found, at baseline, 82.8% reported none or mild dry mouth during the day. In contrast, our study found only 15.6% rated their dry mouth as none to mild. Sweeney [14] also found 80% had either no difficulty or mild difficulty with talking compared with 15.5% in our study. Furthermore, 88.2% had either no or mild impact on eating compared with our study which found only 15.5% reported none or mild interference with eating. These differences may reflect the small sample used in the Sweeney study but may also reflect the different methods used to assess dry mouth, with Sweeney using a 7-point Likert scale (response 0–6) and reducing this to three categories: no problem (0), mild problem (1–3) and severe problem (4–6).

Nearly all patients reported needing to use things to keep their mouth moist, with most patients using a range of both pharmacological and non-pharmacological items. Drinks were the commonest single method used to alleviate dry mouth and evidence from free text suggested water was as effective.

Salivary substitutes replace oral moisture only and need to be taken very regularly including during meals. Salivary stimulants or sialogogues such as pilocarpine, bethanechol and cevimeline improve salivary flow by agonist action at muscarinic cholinergic receptors. Pilocarpine functions primarily as a muscarinic-cholinergic agonist with mild beta-adrenergic activity and is licenced for dry mouth following radiotherapy for head and neck cancer and in Sjogren’s syndrome [24].

A systematic review considered the effectiveness of non-systemic topical interventions in dry mouth from a range of aetiologies [25]. Evidence was found to be limited. A systematic review to determine the effectiveness of pharmacological and non-pharmacological interventions in treating dry mouth in advanced cancer patients (excluding dry mouth caused specifically by radiotherapy, surgery, graft versus host disease and autoimmune diseases) identified only three randomised controlled trials, the majority of which were not statistically significant [13]. The review highlighted the paucity of the evidence base. A recent feasibility study of pilocarpine in advanced cancer patients found that the treatment was unacceptable to most patients due to its side effect profile [18].

Basic mouth care can be effective in managing dry mouth. The spontaneous improvement in dry mouth in the palliative care studies could be related to the greater emphasis on mouth care [13]. A study involving elderly patients in a long-term care facility found that tooth brushing and mouthwash decreased dry mouth and oral tongue plaques [26]. Other simple measures such as taking sips of water found in our study can be effective for providing symptomatic relief.

Nearly half of all patients reported that they had other concerns that had not been identified in the questionnaire. The most common concern was around associated dryness of lips, throat and nasal passages. Simply addressing the dry mouth is often not enough. Maintaining moist lips and nasal passages appears to be important for patients and can be helped by the administration of simple water-based gels and ensuring humidification [23, 27]. Another common concern was around dysphagia and the perception of dysphagia. This can have effects on patient’s diet as well as impact on the fluid intake, further contributing to the symptom of dry mouth [28]. Many patients reported a disturbance in sleep. Simple attention to ensuring a drink is by the bedside of patients can help minimise disturbance and may result in better quality sleep. Almost half of the patients who reported to not ‘usually feel dry’ reported that the dry mouth was a new or recent occurrence in hospital. The association of dry mouth with the commencement of medications was also highlighted. Therefore, reviewing their effectiveness and examining the potential for dose reduction or discontinuation may lead to improvements in dry mouth without compromising control of other symptoms.

In a study in an advanced cancer population [16], the mean number of medications was 2 (range 0–4). We found that the mean number of medications was 3.9 (SD 2.4). This could reflect more medication burden in a palliative care population. Typical medications in our study included opioids, corticosteroids, anti-emetics and benzodiazepines. These were similar to Agar [11].

Of patients who had a preference as to the question or measure which was most relevant to their dry mouth, ‘numeric rating scale’ and ‘interference with talking’ were the most commonly reported. A useful assessment tool for dry mouth could incorporate both of these measures. As the responses patients provided for the VRS scale were consistent with the NRS scale, NRS on its own could be used as a measure for severity. In studies measuring pain, NRS has been found to have higher compliance rates, better responsiveness and ease of use compared with both VRS and VAS [29]. We have not found any studies that have asked patients additional information unspecific to their dry mouth or of self-management strategies. Additionally, our questionnaire assessed what patients thought about the questionnaire itself. In this study, the free text proved to be a valuable addition to assess patient’s concerns and would further enhance an assessment tool for dry mouth in the palliative care population.

Sialometry was not used in our study. From our review of objective measurements used in the measurement of dry mouth, salivary collection was not routinely undertaken in the palliative care population [15–17]. Of those used, procedures were limited and the results were not statistically significant [14]. From a search of the use of sialometry in other populations, it was discovered that some patients who complain of dry mouth do not demonstrate reduced flow rates and conversely, some individuals demonstrate an objective decrease in the flow of saliva but do not complain of oral dryness [30, 31]. Furthermore, Davies [4] found that the relationship between dry mouth and resting whole salivary flow rate (UWSFR) was a relatively sensitive (sensitivity 85%) but a non-specific (specificity 30%) method of detecting xerostomia. Similarly, the relationship between dry mouth and stimulated whole salivary flow rate (SWSFR) is a relatively specific (specificity 78%) but insensitive (sensitivity 47%) method for detecting xerostomia. Based on these findings, the authors suggested that there is no indication for the routine measurement of salivary flow rates in the clinical assessment of patients complaining of dry mouth. Furthermore, we did not want to include a burdensome procedure for the palliative care patients in the study.

Our study was reliant on routinely collected patient data. Different measures were collected which limited the ability to compare data. We used questions taken directly from different assessment tools and amalgamated them into one questionnaire. Though we recognised at the outset the inconsistency in the wording within the questionnaire, the aim of the study was to assess responses to different dry mouth questions for a palliative care population.

To minimise bias involved in an observational study using face-to-face questioning, the researchers were not part of the usual clinical team.

This was a cross-sectional study of 135 palliative care patients and as far as we can tell the only study of this kind. It highlights the severity of dry mouth for patients in the inpatient, day care, outpatient or community setting. To date, there is no consensus on the most valid approach to assessing dry mouth. This study describes the important factors required in a clinical assessment of dry mouth and reminds clinicians to consider the functional impact as well as the severity of the symptom on the patient.

## Conclusions

Given the prevalence and impact of dry mouth in advanced disease, and the paucity of the existing evidence base, more research is needed if improvements in quality of life are to be realised for these patients. Improving research methodology is most likely to lead to important improvements in clinical care for patients with dry mouth in the context of advanced disease. This study highlights that dry mouth in patients with advanced disease can have a significant negative impact on the day-to-day ability to talk, eat and taste and can interfere with nasal passages, lips, throat, swallowing and sleep. This data can be used to describe what dry mouth means for a palliative care patient and can be used as an outcome measure in managing and assessing dry mouth. Outcomes from this study could help inform future development of an assessment tool for dry mouth in palliative care patients to be used in clinical practice.
